# Can CONUT and PNI Scores Predict Necrotizing Pancreatitis in Acute Pancreatitis Patients Presenting to the Emergency Department?

**DOI:** 10.3390/jcm13195902

**Published:** 2024-10-03

**Authors:** Mehmet Göktuğ Efgan, Zeynep Karakaya, Efe Kanter, Süleyman Kırık, Mustafa Agah Tekindal

**Affiliations:** 1 Faculty of Medicine Department of Emergency Medicine, Izmir Katip Çelebi University, 35620 Izmir, Turkey; goktugefgan@gmail.com (M.G.E.); zeynepkarakaya76@hotmail.com (Z.K.); efekanter@hotmail.com (E.K.); 2 Faculty of Medicine Department of Biostatistics, Izmir Katip Çelebi University, 35620 Izmir, Turkey; matekindal@gmail.com

**Keywords:** acute pancreatitis, necrotizing pancreatitis, CONUT score, PNI score, prognosis, emergency department

## Abstract

**Background and Objectives:** Acute pancreatitis, characterized by pancreatic inflammation, poses significant morbidity and mortality worldwide, with varied etiologies including gallstones, alcohol, and certain medications. Necrotizing pancreatitis represents a severe form of parenchymal damage with considerable impact on patient quality of life. Early identification of necrotizing pancreatitis is crucial for timely intervention and improved outcomes. The aim of this study was to investigate the usability of CONUT and PNI scores as prognostic indicators. **Materials and Methods:** We conducted a retrospective observational study involving patients presenting to the emergency department with acute pancreatitis between January 2020 and October 2023. The Controlling Nutritional Status (CONUT) score and Prognostic Nutrition Index (PNI) were calculated from serum biomarkers to assess nutritional status. Patients were categorized into necrotizing and nonnecrotizing pancreatitis groups, and the utility of CONUT and PNI scores in predicting necrotizing pancreatitis was evaluated. **Results:** A total of 339 patients were included, with 8.26% diagnosed with necrotizing pancreatitis. CONUT and PNI scores significantly differed between necrotizing and nonnecrotizing groups, with higher CONUT scores and lower PNI scores observed in the necrotizing group. Receiver operating characteristic (ROC) curve analysis revealed significant predictive value of CONUT and PNI scores for necrotizing pancreatitis, with cutoff values of >5 and ≤34, respectively. **Conclusions:** CONUT and PNI scores demonstrate promise in predicting necrotizing pancreatitis in patients admitted to the emergency department with acute pancreatitis. Additionally, these scores may serve as prognostic indicators for mortality in acute pancreatitis patients. Early identification using CONUT and PNI scores could facilitate timely intervention, potentially reducing mortality and morbidity in this patient population.

## 1. Introduction

Acute pancreatitis is an inflammatory condition of the pancreas characterized by the premature activation of pancreatic enzymes and leading to pancreatic tissue damage and systemic inflammation. While the leading cause is typically gallstone-induced obstruction of the pancreatic duct, other factors such as alcohol consumption, endoscopic retrograde cholangiopancreatography (ERCP), and certain medications can also incite inflammation in the pancreatic acinar cells [1,2,3]. After the diagnosis is made, depending on whether there is necrotizing pancreatitis or not, surgery may be required for necrotizing pancreatitis, albeit rarely. Necrotizing pancreatitis is the most severe form of tissue injury in acute pancreatitis, characterized by the death of pancreatic and/or peripancreatic tissue. Treatment decisions, whether endoscopic or surgical, depend on the presence of necrosis. However, interventions should be delayed or avoided when possible, with endoscopic approaches such as endosonographic transgastric drainage and necrosectomy considered the first-line treatment. Contrast-enhanced computed tomography remains the gold standard for diagnosing necrotizing pancreatitis. Imaging findings in necrotizing pancreatitis may not always be apparent during the early stages, such as upon emergency department admissions. Typically, it takes around 72 h for complications to become visible. While contrast-enhanced CT is commonly used, MRI offers superior sensitivity for detecting necrosis earlier. Therefore, there is a need for reliable laboratory parameters to predict necrotizing pancreatitis in the emergency department [4].

The Controlling Nutritional Status (CONUT) score has emerged as a readily accessible tool for nutritional screening, evaluating patients’ nutritional status through serum albumin level, total cholesterol level, and total lymphocyte count [5]. Subsequent studies have underscored its clinical significance in predicting long-term outcomes among patients with gastrointestinal and hepatopancreatobiliary cancers [6,7,8].

The Prognostic Nutrition Index (PNI), calculated by serum albumin concentration and peripheral blood lymphocyte count, can evaluate perioperative nutritional conditions and postoperative complications in patients with malignant gastrointestinal system tumors [8,9]. Studies indicate the prognostic importance of the PNI outside gastrointestinal system tumors [10,11,12]. In a recent study, it was stated that PNI values can be used as prognostic indicators in patients with acute pancreatitis [9].

Although studies report that CONUT and PNI scores can be used as prognosis indicators in acute pancreatitis, no study in the literature has investigated whether they can be used for the early detection of necrotizing acute pancreatitis, an important complication of acute pancreatitis. Our aim in this study was to determine whether CONUT and PNI scores can predict necrosis in acute pancreatitis and to assess their usability as prognostic indicators.

## 2. Materials and Methods

### 2.1. Study Design

This was a retrospective observational study. The study included patients admitted to the emergency department between 1 January 2020 and 1 October 2023. Local ethics committee approval was obtained before starting the study.

### 2.2. Study Population

Our study included patients aged 18 years and older who presented to the emergency department and were diagnosed with acute pancreatitis. Among these patients, we excluded patients with additional pathologies that may cause abdominal pain, patients with additional infections, pregnant or breastfeeding women, patients with missing data, patients with a history of malignancy, patients with sepsis, patients with acute renal failure, patients with hepatic failure, and patients with a history of anti-inflammatory or immunosuppressive drug use. Patients referred to an external center and for whom no outcome information was available were also excluded.

### 2.3. Study Sample Size

The positive group represents necrotizing pancreatitis. A sample size of 300 from the positive group and 339 from the negative group provides 80.19% power to detect a difference of 0.05 in the area under the ROC curve (AUC). This comparison is made between a null-hypothesis AUC of 0.80 and an alternative-hypothesis AUC of 0.85 using a two-sided z-test with a significance level of 0.05. The data consist of discrete (rating scale) responses, and the AUC is calculated between false-positive rates of 0 and 1. The standard deviation of the responses in the negative group is equal to that of the positive group, with a ratio of 1.

### 2.4. Data Collection

Patient records were accessed on the hospital information management system to determine the patients to be included in the study. ICD10 diagnosis codes K85, K85.8, and K85.9 were searched in the hospital’s information management system to identify patients diagnosed with acute pancreatitis in the emergency department within the specified date range, and the results were confirmed by scanning the patient files. A total of 418 patients were identified. Of these patients, 29 were excluded because of a history of malignancy, 31 because of missing data, 14 because of liver failure, 3 because of a history of hematological disease, 2 because of pregnancy, and 1 because they were receiving immunosuppressive treatment. The remaining 339 patients were included in the study. Age, gender, laboratory data, imaging data, length of hospitalization, mortality, and comorbidities of all patients were recorded in the data recording form for statistical analysis. The patients were segregated into two categories: those with necrotizing acute pancreatitis and those with nonnecrotizing acute pancreatitis. The study aimed to assess the efficacy of the computed CONUT and PNI scores in distinguishing between these two groups. Furthermore, the study analyzed whether CONUT and PNI scores could serve as prognostic indicators for all patients with acute pancreatitis.

### 2.5. Outcomes

The primary aim of this study is to examine the predictive role of CONUT and PNI scores in predicting necrotizing pancreatitis. The secondary outcome evaluated whether CONUT and PNI scores can be prognostic indicators in patients with acute pancreatitis.

### 2.6. Calculation of Data

The study performed calculations using hemogram and biochemistry results for each case. CONUT and PNI scores were calculated.

- CONUT score is calculated as albumin score + lymphocyte score + total cholesterol score.

**Parameter**

**>3.5 or >1600 or >180**

**3.0–3.4 or 1200–1599 or 140–179**

**2.5–2.9 or 800–1199 or 100–139**

**<2.5 or <800 or <100**
Albumin (g/dL)0246Lymphocytes (/mL)0246Total Cholesterol (mg/dL)0246

Total score: 0–1 = normal.

2–4 = medium

5–8 = moderate.

9–12 = serious.

- PNI score: calculated as (10× albumin) + (0.005× lymphocytes).

Total score: >38 = normal.

35–38 = medium.

<35 = serious.

### 2.7. Statistical Analysis

After the data collection process, the data were digitized and analyzed statistically. SPSS Statistics for Windows, Version 27.0. (IBM Corp., Armonk, NY, USA) was used for all analyses. Any *p* value less than 0.05 was considered significant, and all analyses were performed within a 95% confidence interval. Descriptive statistics are presented as frequency, percentage, mean, standard deviation, median, minimum, and maximum values. The Shapiro–Wilk test was used to test normality assumptions, skewness, kurtosis values, and Q–Q plots. The participants’ data were compared with the independent-sample *t*-test when they fit the normal distribution and with the Mann–Whitney U test when they did not.

## 3. Results

A total of 339 patients were included in the study, and the mean age was 61.35 ± 15.3 years. While 28 (8.26%) of the patients had necrotizing acute pancreatitis, 311 (91.74%) were in the nonnecrotizing acute pancreatitis group. In sum, 17 (5.01%) patients were hospitalized in intensive care units and 30 (8.8%) patients resulted in exitus. Descriptive statistics of the patients are presented in Table 1.

Statistically significant differences were observed between the necrotizing and nonnecrotizing pancreatitis groups when comparing CONUT and PNI scores. In the necrotizing group, the CONUT score averaged 5.43 ± 3.18, whereas in the nonnecrotizing group, it was 3.44 ± 2.58. This difference in CONUT scores between the two groups was statistically significant, with higher scores seen in the necrotizing group. Conversely, the PNI score averaged 38.71 ± 8.3 in the necrotizing group and 42.48 ± 7.68 in the nonnecrotizing group. The PNI score was significantly lower in the necrotizing group compared to the nonnecrotizing group. Table 2 provides a comparison of the CONUT and PNI scores between the groups.

**Figure 1:**
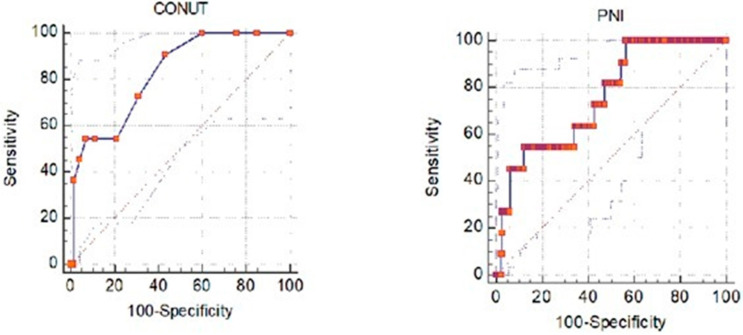
ROC curves for CONUT and PNI scores to predict necrotizing acute pancreatitis.

ROC curve analysis was performed for CONUT and PNI scores to predict necrotizing acute pancreatitis in patients diagnosed with acute pancreatitis. CONUT and PNI scores were statistically significant in predicting necrotizing acute pancreatitis. The cutoff value for the CONUT score was found to be >5. Scores above 5 are more likely to be necrotizing. This cutoff value’s area under the curve was calculated as 0.681, sensitivity 50.00%, and specificity 78.78%. The cutoff value for the PNI score was calculated as ≤34. Scores below this cutoff value are more likely to be necrotizing. For this cutoff value, the area under the curve was calculated as 0.651, sensitivity 39.29%, and specificity 87.46%. ROC curve analysis of CONUT and PNI measurements for necrotizing and nonnecrotizing patients are presented in Table 3 and Figure 1.

As shown in Table 4, the Cox regression results for CONUT and PNI values were statistically significant. Although there was a small difference between them in terms of hazard ratios, it can be stated that the CONUT value posed a higher risk compared to the PNI value.

As shown in Table 5, the Cox regression results for CONUT and PNI values were statistically significant. Although there was a small difference between them in terms of hazard ratios, it can be stated that the CONUT value posed a higher risk compared to the PNI value.

**Figure 2:**
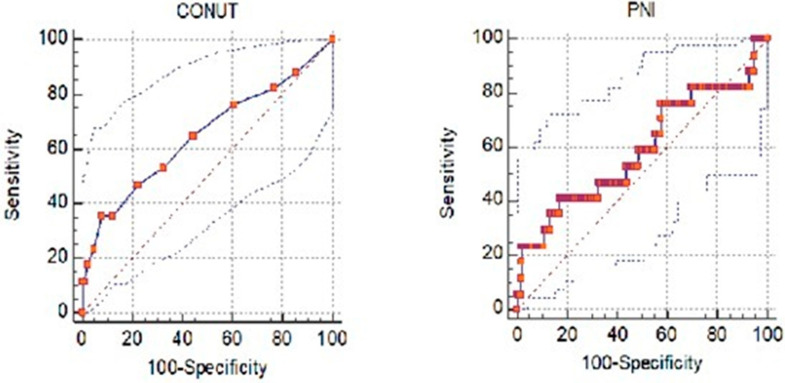
ROC curves for CONUT and PNI scores to predict exitus.

ROC curve analysis was performed for CONUT and PNI scores to predict exitus status in patients diagnosed with acute pancreatitis. The use of CONUT and PNI scores for predicting exitus was statistically significant. The cutoff value for the CONUT score was found to be >3. For scores >3, the probability of exitus was higher. These cutoff values under the curve were calculated as 0.824, sensitivity 90.91%, and specificity 56.63%. The cutoff value for the PNI score was calculated as ≤43.45. Exitus was more likely in scores below this cutoff value. For this cutoff value, the area under the curve was calculated as 0.758, sensitivity as 100.0%, and specificity as 43.69%. ROC curve analysis based on exitus with CONUT and PNI measurements is presented in Table 6 and Figure 2.

**Figure 3:**
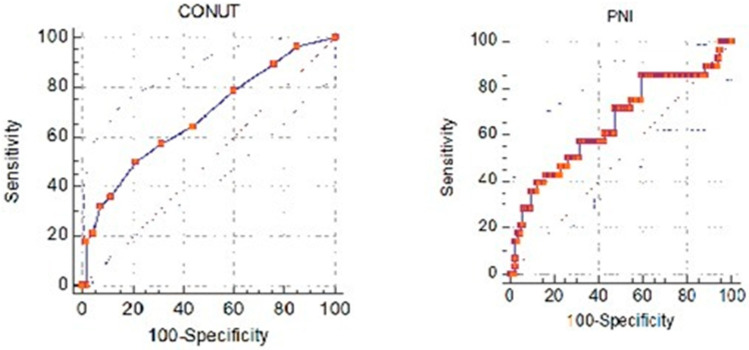
ROC curves for CONUT and PNI scores to predict intensive care unit admission.

ROC curve analysis was performed for CONUT and PNI scores to predict the need for intensive care admission in patients diagnosed with acute pancreatitis. Using the CONUT score to predict the need for intensive care admission was statistically significant. The PNI score could not be used to predict the need for intensive care in patients with acute pancreatitis. The cutoff value for the CONUT score was found to be >7. Scores above 7 indicate a higher need for intensive care admission. For this cutoff value, the area under the curve was calculated as 0.649, sensitivity as 42.86, and specificity as 96.71%. ROC curve analyses based on need for intensive care with CONUT and PNI measurements are presented in Table 7 and Figure 3.

## 4. Discussion

The relationship between clinical and nutritional status in acute pancreatitis patients is unknown. Be Discoveries has reported that disease related malnutrition is commonly observed during hospitalization [13,14]. However, the relationship between nutritional status, especially at the time of emergency department admission, and the clinical course and outcome of the disease has yet to be established. Recent studies have reported that scores such as CONUT and PNI, which are used as indicators of nutritional status, can be used as prognostic indicators in patients with acute pancreatitis [9,15]. It has been reported that changes in these scores are associated with a higher risk of complications. Apart from these scores, there are many other scoring systems available to assess nutritional status. Among them, widely used methods include Nutritional Risk Screening (NRS-2002), Subjective Global Assessment (SGA), the Malnutrition Universal Screening Tool (MUST), the Geriatric Nutritional Risk Index (GNRI), Mini Nutritional Assessment (MNA), and the Nutritional Risk Index (NRI) [16,17,18,19,20]. However, most of these scoring systems include parameters such as body weight and BMI, which limit their use in situations requiring rapid assessment in the emergency department. In contrast, CONUT and PNI scores rely on laboratory values rather than body weight parameters, making them more suitable for use in the emergency setting. However, no studies in the literature have investigated the usefulness of CONUT and PNI scores in predicting necrotizing acute pancreatitis, the most important complication of acute pancreatitis. In this study, we found that CONUT and PNI scores can be used to predict necrotizing acute pancreatitis in acute pancreatitis patients admitted to the emergency department.

Acute pancreatitis is a state of inflammation. Moreover, as necrosis develops, inflammation increases. Inflammation leads to decreased appetite and increased insulin resistance [15], and this is associated with various metabolic processes that prevent the passage of glucose in the blood to the cells [21]. The CONUT score comprises albumin, which serves as a negative acute-phase reactant closely associated with inflammation, lymphocytes, and total cholesterol, which is closely linked to nutritional status. Albumin is a key component that plays a crucial role in nitrogen balance and acts as a nitrogen source during negative nitrogen balance [22]. Overcoming negative nitrogen balance is a primary goal in acute pancreatitis treatment, emphasizing the importance of initiating early enteral nutrition [23]. This significant aspect of albumin underscores its relevance to the relationship between CONUT and PNI scores and prognosis in emergency department-admitted patients. Given its reflection of nutritional status, albumin is likely intricately linked to inflammation. Previously, Wang et al. [23] reported that albumin and total cholesterol were poor prognostic indicators for in-hospital mortality in acute pancreatitis patients. Our study has shown that the use of the CONUT score can be used to predict necrotizing acute pancreatitis in patients with increased inflammation due to acute pancreatitis. A CONUT score exceeding 5 suggests a likelihood of necrotizing acute pancreatitis. Sensitivity was 50.00%, specificity was 78.78%, and the AUC value was 0.681. Although the results were usable, it should be noted that the power was limited. The PNI score is composed of albumin and lymphocyte components, reflects nutritional status, and is associated with inflammation. This study concluded that the PNI score can predict necrotizing acute pancreatitis in patients admitted to the emergency department. For the PNI score, the AUC value was 0.651, with a sensitivity of 39.29%, specificity of 87.46%, and a cutoff value of 34. Although the result demonstrates statistical significance, its strength is limited. These two scores, which consist of components reflecting inflammation, can be used to predict the diagnosis of necrotizing acute pancreatitis in patients presenting to the emergency department. In this way, early detection of an important complication of acute pancreatitis and early initiation of necessary treatment can be ensured.

This study found that the first calculated CONUT score can predict the need for intensive care and mortality in patients with acute pancreatitis admitted to the emergency department. We calculated that CONUT scores above 3 predicted mortality with 90.91% sensitivity and 56.63% specificity. Akkuzu et al. evaluated CONUT and PNI scores as prognosis indicators in patients with acute pancreatitis [9] and reported that CONUT scores were higher in patients with mortality. They argued that high CONUT scores were associated with poor prognosis. Akkuzu et al. [9] also reported that the need for intensive care increased as the CONUT score increased. In this study, the CONUT score predicted the need for intensive care with a sensitivity of 42.86% and a specificity of 96.71% at scores above 7. Although this result is not very strong, it is compatible with the literature. Shi et al. reported that high CONUT scores were associated with mortality in their study [14], in which they investigated the feasibility of using CONUT scores to predict poor short-term prognosis in acute pancreatitis patients. This study emphasizes the conclusion that high CONUT scores are associated with mortality in patients with acute pancreatitis, in accordance with the literature.

PNI score is associated with poor prognosis in patients with acute pancreatitis. In this study, it was found that low PNI scores can predict mortality, but their use in predicting the need for intensive care is not appropriate. Patients with a PNI score ≤ 43.45 had a higher probability of exitus with a sensitivity of 100% and a specificity of 43.69%. The PNI score was also reported to be associated with poor prognosis in the previously reported study by Akkuzu et al. Akkuzu et al. [9] found that low PNI scores were associated with intensive care unit hospitalization. Although the two studies reached similar results in predicting mortality, there are different results regarding the need for intensive care. This difference may be due to the difference in the patient population or due to nondiagnostic reasons that may arise in the decision to hospitalize patients in intensive care. However, the common point is that a decreased PNI score indicates a poor prognosis. Poor nutritional score at baseline indicates a poor prognosis in patients with acute pancreatitis, for whom we have found that nutritional problems in treatment affect patient outcomes.

The Ranson and Glasgow scoring systems have traditionally played an important role in predicting the prognosis of acute pancreatitis [24,25]. However, in our study, we were unable to apply these scoring systems due to the unavailability of the LDH parameter in our emergency department. Instead, our study focused on the CONUT and PNI scores, which have shown potential effectiveness in predicting the prognosis of acute pancreatitis.

The most important limitation of this study is its single-center and retrospective design. The second important issue was that we needed to have information about the nutritional status of the patients before their emergency department admission. In addition, although conditions that may affect nutrition and change the scores before hospital admission are accepted as exclusion criteria, unknown conditions may always be present. Moreover, this may have affected our results. In addition, our limited number of patients is also a significant limitation. It would be useful to repeat the study with larger samples.

## 5. Conclusions

CONUT and PNI scores may predict necrotizing acute pancreatitis in patients with acute pancreatitis admitted to the emergency department. In addition, these scores can be used to predict mortality in patients with acute pancreatitis. Therefore, we recommend the use of both scores, although the CONUT score appears superior in comparative analysis to prevent mortality and morbidity in patients admitted to the emergency department.

## Figures and Tables

**Table 1:** Descriptive statistics of patients.

	Statistics
**Number of Days in Hospital**	8.61 ± 10.22
**Age**	61.35 ± 15.3
**Gender**	
Women	191 (56.34%)
Man	148 (43.66%)
**Necrotizing Pancreatitis**	
No	311 (91.74%)
Yes	28 (8.26%)
**Respiratory Rate**	14.5 ± 2.9
**Body Temperature**	36.77 ± 0.86
**Pulse**	85.07 ± 15.44
**BUN**	19.01 ± 9.53
**Creatinine**	1.05 ± 0.77
**CRP**	66.29 ± 78.51
**Albumin**	35.07 ± 5.65
**Total cholesterol**	167.86 ± 53.38
**Lipase**	1891.25 ± 2732.47
**Total Bilirubin**	2.06 ± 2.87
**Direct Bilirubin**	1.2 ± 2.04
**Indirect Bilirubin**	0.86 ± 0.93
**AST**	124.23 ± 161.18
**ALT**	131.4 ± 162.22
**Leukocytes**	11.9 ± 5.39
**Neutrophil**	9.51 ± 4.96
**Lymphocytes**	1.42 ± 0.83
**CONUT score**	3.6 ± 2.68
**PNI score**	42.17 ± 7.79
**Outpatient/Service/ICU**	
Outpatients	19 (5.6%)
Service	303 (89.38%)
ICU	17 (5.01%)
**Exitus/Discharged**	
Discharged	309 (91.15%)
Exitus	30 (8.8%)

**Table 2:** Comparison of CONUT and PNI scores between the groups.

	Necrotizing Pancreatitis		Test Statistics	*p*
	No	Yes		
**CONUT**	3.44 ± 2.58	5.43 ± 3.18	−3.191	**0.001**
**PNI**	42.48 ± 7.68	38.71 ± 8.3	−2.642	**0.008**

**Table 3:** Cutoff scores, AUC value, sensitivity, selectivity, and statistical significance of CONUT and PNI measurements for necrotizing and nonnecrotizing patients.

Test Result Variables	*Cutoff*	AUC	Std. Error	*p*	Asymptotic 95% Confidence Interval		*Sensitivity*	*Specificity*
					Lower Bound	Upper Bound		
CONUT	>5	0.681	0.056	**0.001**	0.628	0.730	50.00	78.78
PNI	≤34	0.651	0.060	**0.012**	0.597	0.701	39.29	87.46

**Table 4:** Cox regression analysis of CONUT and PNI for necrotizing pancreatitis groups (with hospital length of stay).

	*β*	SE	Wald	df	Sig.	Exp (*β*)	95.0% CI for Exp (*β*)	
							Lower	Upper
CONUT	0.095	0.120	0.620	1	0.040	1.099	1.068	1.392
PNI	0.051	0.048	1.119	1	0.035	1.052	1.003	1.157

**Table 5:** Cox regression analysis of CONUT and PNI for exitus groups (with hospital length of stay).

	*β*	SE	Wald	df	Sig.	Exp (*β*)	95.0% CI for Exp (*β*)	
							Lower	Upper
CONUT	0.107	0.117	0.838	1	0.037	1.113	1.014	1.399
PNI	0.054	0.046	1.357	1	0.029	1.055	1.003	1.156

**Table 6:** Cutoff scores, AUC values, sensitivity, selectivity, and statistical significance of CONUT and PNI measurements based on exitus.

Test Result Variables	*Cutoff*	AUC	Std. Error	*p*	Asymptotic 95% Confidence Interval		*Sensitivity*	*Specificity*
					Lower Bound	Upper Bound		
CONUT	>3	0.824	0.057	**0.001**	0.778	0.864	90.91	56.63
PNI	≤43.45	0.758	0.069	**0.002**	0.707	0.804	100.0	43.69

**Table 7:** Cutoff scores, AUC values, sensitivity, selectivity, and statistical significance of CONUT and PNI measurements based on intensive care need.

Test Result Variables	*Cutoff*	AUC	Std. Error	*p*	Asymptotic 95% Confidence Interval		*Sensitivity*	*Specificity*
					Lower Bound	Upper Bound		
CONUT	>7	0.649	0.081	**0.039**	0.595	0.660	42.86	96.71
PNI	≤35	0.592	0.084	0.275	0.537	0.644	41.18	82.92

## Data Availability

The original contributions presented in the study are included in the article, further inquiries can be directed to the corresponding authors.
